# Effects of filial piety belief on cyberbullying perpetration of Chinese university students

**DOI:** 10.3389/fpsyg.2022.1018449

**Published:** 2022-12-07

**Authors:** Hua Wei, Lijun Lu, Meiting Liu

**Affiliations:** ^1^Normal College, Qingdao University, Qingdao, China; ^2^School of Educational Science, Xinyang Normal University, Xinyang, China; ^3^Department of Social Research, University of Turku, Turku, Finland

**Keywords:** traditional Chinese culture, dual filial piety model, cyberbullying perpetration, relatedness need satisfaction, Chinese university students

## Abstract

Cyberbullying has become a worldwide phenomenon. Although the topic has drawn decent academic attention and many studies have been conducted on Chinese samples, variable interests in these studies have not captured the thinking and behavioral characteristics of Chinese people. Based on the dual filial piety model and self-determination theory, this study examined the effect of filial piety belief on cyberbullying perpetration and tested the mediation of relatedness need satisfaction. A total of 856 university students completed the questionnaires, including dual filial piety scale, relatedness need satisfaction scale and cyberbullying perpetration scale. The regression results found that reciprocal filial piety negatively predicted and authoritarian filial piety positively predicted cyberbullying perpetration. The SEM results showed that reciprocal filial piety and authoritarian filial piety impacted cyberbullying perpetration through the mediating effect of relatedness need satisfaction. Reciprocal filial piety was positively while authoritarian filial piety negatively correlated with relatedness need satisfaction and relatedness need satisfaction was negatively correlated with cyberbullying perpetration. The results provide a new position to understand the effect of family factors on cyberbullying perpetration by placing the topic within traditional Chinese family value.

## Introduction

Although the rapid development of the Internet has largely facilitated interpersonal communication, serious social conflicts have emerged among net citizens, for example, cyberbullying. Cyberbullying perpetration is defined as the behavior that one party inflicts aggressive behaviors (e.g., verbal and textual insults) toward another party through social networking tools or network communication tools (Patchin and Hinduja, 2006; Tokunaga, 2010; Kowalski and Limber, 2013; Kowalski et al., 2014). Today, cyberbullying has become a worldwide phenomenon (Zhou et al., 2013; Kowalski et al., 2014; Zurcher et al., 2018). As a result, cyberbullying victims have more mental and behavioral issues than their school peers. For example, adolescents who are bullied may experience more negative emotions, including anxiety, depression, loneliness and even suicidal intent (Larrañaga et al., 2016; Chu et al., 2018; Iyanda, 2021). The victims also have more problematic behaviors, including substance abuse, truancy and dropping out of school (Kowalski et al., 2014; Kim et al., 2019). Considering the obvious harm, investigating the factors generating cyberbullying perpetration is needed. Although the topic has drawn decent academic attention and many studies have been conducted on Chinese samples (Lam et al., 2013; Yang et al., 2018; Fan et al., 2019; Wang et al., 2019a,b), variable interests in these studies, for example, narcissism, childhood maltreatment, interparental conflict, exposed to violent video games, have not captured the thinking and behavioral characteristics of Chinese people.

Despite the rapid advancement of modern society, traditional culture still has an important impact on Chinese people. In specific, filial piety as a particular norm and value shapes the thinking and action of Chinese descendants as well as influencing people from eastern Asia and south-eastern Asia (Chappell and Kusch, 2007; Tan et al., 2018; Bedford and Yeh, 2019). Filial piety is a moral norm which specifies what children should do to respect and care for their parents. A previous study has shown that such moral norm in a family can be expanded to individual morality in more public domains, for instance, decreasing aggressive behaviors (Yeh, 2006). Thus, it is of theoretical and practical significance to examine cyberbullying perpetration in relation to filial piety. Based on the dual filial piety model (Yeh and Bedford, 2003), this study is to examine the impact of filial piety belief on cyberbullying and the influential mechanism underlying this association.

### Filial piety belief and cyberbullying perpetration

Filial piety is one of the core idea of Confucian ethics, underlining moral norms concerning material and emotional aspects of the parent–child relationship (Bedford and Yeh, 2019). The composition of the Chinese character (filial piety, pronouncing “xiao”), formed by a component character representing the aged on the top and a component character representing the child on the bottom, suggests both a status difference between children and parents and an implication that children should support parents. Filial piety not only regulates the morality in a family but also provides the moral foundation for social norms so as to build a stable society. Just as what Confucius said, “filial piety begins at serving parents, then serves monarch, and ends with establishing self-position in society.” Further, filial piety belief is concerned with to which degree individuals accept filial piety as a principle to form their family morality, or how important they think of the idea regarding being a good daughter/son.

Given that previous researchers disagree on whether filial piety belief is beneficial and both sides have empirical support (Ho and Lee, 1974; Ishii-Kuntz, 1997), some researchers have constructed a dual filial piety model to elucidate the disagreements (Yeh and Bedford, 2003; Bedford and Yeh, 2019). The model is comprised of two attributes, maintaining that reciprocal filial piety (RFP) has more positive outcomes, whereas authoritarian filial piety (AFP) has more negative outcomes.

According to the dual filial piety model, we assume that RFP may reduce the likelihood of individual perpetrating cyberbullying, whereas AFP increase this likelihood. Net citizens get to know members from out-groups holding different backgrounds, experiences and opinions, because the internet has largely expanded the range of social interaction. Compared with socializing with in-group members, socializing out-group members is more likely to encounter conflicts and aggression (Forbes et al., 2011). Individuals who hold filial piety belief may handle this situation differently. Equality is the essence of RFP, which emphasizes the care and understanding between parents and children. Continuously influenced by this equal mode, adolescents may have better perspective taking, higher openness and a stronger tendency toward common human decency (Yeh and Bedford, 2003; Yeh, 2006; Leung et al., 2010; Bedford and Yeh, 2019). Adolescents with these traits may have higher tolerance to the difference between groups, which may reduce conflicts and cyberbullying perpetration.

However, hierarchy is the essence of AFP, which underlines children’s unconditional submission to parents. Chronically influenced by this hierarchical mode, adolescents may have worse perspective taking, lower openness and a stronger tendency toward particularism (Yeh and Bedford, 2003; Yeh, 2006; Leung et al., 2010; Bedford and Yeh, 2019). Correspondingly, adolescents who hold AFP may have a strong preference for in-group members and thus a strong exclusion of out-group members, leading to an increase in conflicts and cyberbullying perpetration. In addition, previous studies have found that RFP negatively predicts aggressive behaviour and AFP positively predicts aggressive behaviour (Yeh, 2006).

Thereby, we propose Hypothesis1: RFP is negatively correlated with cyberbullying perpetration, and AFP is positively correlated with cyberbullying perpetration.

### Mediation effect of relatedness need satisfaction

According to the self-determination theory, the satisfaction of basic psychological needs is vital to individual development and thus unsatisfied needs can induce problematic behaviors (Deci and Ryan, 2000; Ryan and Deci, 2017). Many recent empirical studies have demonstrated the significant association between the satisfaction of basic psychological needs and aggressive behaviors (Hein et al., 2015; Fousiani et al., 2016; Ryan and Deci, 2017; Choe and Read, 2019). Researchers have also found that the satisfaction of basic psychological needs can mediate the association between family factors and aggressive behaviors (Fousiani et al., 2016; Choe and Read, 2019). Therefore, we expect that relatedness need satisfaction can be a possible mediating variable underlying the association between filial piety belief and cyberbullying perpetration.

To begin with, we consider that filial piety belief is positively correlated with relatedness need satisfaction: RFP increases relatedness need satisfaction, whereas AFP reduces relatedness need satisfaction. In order to achieve the goal of genuinely caring for parents that RFP emphasizes, individuals need to understand their parents and put themselves in their parents’ shoes. Therefore, individuals may develop perspective taking and social competence during the process of caring for parents (Yeh and Bedford, 2003; Leung et al., 2010). In contrast, AFP underlines children’s unconditional obedience to their parents. In other words, children only need to obey their parents’ orders, understand their parents’ ostensible intentions so that do not need to empathize with their parents. Therefore, perspective taking and social competence would be difficult to be developed (Yeh and Bedford, 2003; Leung et al., 2010). Individuals who score high on perspective taking and perceived social competence have better interpersonal relationships and are less likely to feel lonely, thus predicting more relatedness need satisfaction (Schröder-Abé and Schütz, 2011; Haugen et al., 2013; Peterson et al., 2015).

In addition, we assume that relatedness need satisfaction is negatively correlated with cyberbullying perpetration. Self-determination theory maintains that relatedness, autonomy and competence are the three basic psychological needs of human beings (Deci and Ryan, 2000; Ryan and Deci, 2017), and thus the satisfaction of the three needs is important for individual development. According to this theory, if basic needs are met, individuals can effectively function and develop in a healthy manner, whereas individuals would exhibit mental morbid and unsatisfactory functional status if the basic needs are unsatisfied (Deci and Ryan, 2000; Ryan and Deci, 2017). More seriously, the dark side of the human behavior (dishonesty, lack of empathy, and aggression) will emerge if the basics needs are unfulfilled. For example, bullying behaviour in high-schools are more common in areas where individuals’ basic needs are not met (Hein et al., 2015).

Relatedness need is one of three basic needs. Recent research has shown that relatedness need satisfaction and cyberbullying perpetration are negatively associated (Fousiani et al., 2016). In addition, the association between relatedness need satisfaction and cyberbullying perpetration could be inferred from the perspective of the social-information processing model, which argues that hostile cognition is an important factor in the increased aggression (Crick and Dodge, 1994). A lack of relatedness need satisfaction may contribute to individual loneliness (Wei et al., 2015), and the increase in loneliness lead to an increase in hostile cognition (Spithoven et al., 2017), consequently resulting in cyberbullying perpetration.

Thereby, we propose H2: Filial piety belief influences cyberbullying perpetration through the mediating role of relatedness need satisfaction. RFP positively affects relatedness need satisfaction, whereas AFP negatively affects relatedness need satisfaction. Relatedness need satisfaction has a negative impact on cyberbullying perpetration.

To sum up, based on the dual filial piety model and self-determination theory, this study is to examine the effect of filial piety belief on cyberbullying perpetration and test the mediating role of relatedness need satisfaction.

## Materials and methods

### Participants

We recruited university students (*n* = 895) from three universities in a city in central China during the spring semester in 2019. After eliminating invalid answers, we received completed questionnaires from 856 students (95.6%). We deleted missing data, deleting in the order of 3 gender, 3 age, 21 RFP, 1 AFP and 11 cyberbullying perpetration. The final sample consisted of 311 (36.3%) male participants and 545 (63.7%) female participants, *M*_age_ = 19.94, *SD* = 1.38.

### Procedure

Before investigation, we received permission to conduct this survey from the Ethical Committee for Scientific Research in our institution and we also sought consent from participants about the research use of their answers. This study was conducted by trained researchers majoring in psychology. Before collecting data, we ensured these university students fully understand the whole process of this survey, such as elaborating on the instructions, explaining obscure items to some participants, and checking participants’ completeness in the questionnaire. Voluntariness and confidentiality were clearly explained in this survey. Participants were informed that they could refuse or discontinue the questionnaire at any time without penalty; they were also asked not to write their name or student number on their questionnaires for confidentiality. Afterwards, participants completed a questionnaire that gauged their demographic characteristics, RFP, AFP, relatedness need satisfaction and cyberbullying perpetration experiences in the recent half year.

### Measures

#### Cyberbullying perpetration

Cyberbullying perpetration inventory contains 6 items that describe 6 forms of cyberbullying perpetration behaviors, such as “tease someone through the Internet (Wechat, tencent QQ, emails, messages, forum, social media, etc.)” and “threaten someone through the Internet” (Lam and Li, 2013). A response set with a rating from 0 to 6 corresponding to a range of 0 times to 6 times or more cyberbullying perpetration experience in the recent half year. We computed the mean scores of the 6 items, with higher values representing higher incidence rates of cyberbullying perpetration. In this study, the Cronbach alpha coefficient is 0.85.

#### Filial piety belief

The filial piety belief scale was developed from the original Filial Piety Scale (Yeh and Bedford, 2003) and appropriately adapted for mainland and Chinese adolescents. The scale consists of 10 questions, including 5 questions for RFP and 5 questions for AFP. Examples of items measuring RFP include, ‘When parents are not happy, children should talk to their parents, understand and comfort them’; AFP items include, “No matter what parents do, children should do it immediately.” Participants’ responses were made on a five-point scale ranging from 1 (completely disagree) to 5 (completely agree). In this study, the Cronbach alpha coefficients were 0.88 for RFP and 0.81 for AFP.

#### Relatedness need satisfaction

Relatedness need satisfaction was evaluated by the 3-item subscale of a basic need satisfaction scale [Neubauer and Voss, 2016; e.g., “I felt close and connected with other people who are important to me.” Answers were given on a seven-point scale, ranging from 1 (completely disagree) to 7 (completely agree)]. The higher the scores, the higher the relatedness need satisfaction. The Needs Satisfaction Scale has been shown to be reliable and valid in previous studies involving a Chinese sample (Wang et al., 2017; Hui et al., 2019; Novak et al., 2021; Xu et al., 2021). Cronbach’s alpha for the relatedness need satisfaction scale in the current study was 0.85.

### Statistical analyses

We used SPSS 18.0 to analysis the data and performed three statistical procedures as follows.

First, descriptive analyses were taken to understand the general condition of the variables in the current study. Further, Pearson’s correlation analyses were used to investigate the potential associations among the independent variables (RFP and AFP), the mediator (relatedness need satisfaction) and dependent variables (cyberbullying perpetration). Second, linear regressions were performed to compare the different effects of RFP and AFP on cyberbullying perpetration, simultaneously controlling for gender and age. Third, we used SEM to test the mediating effect involving all variables using AMOS 21.

## Results

### Descriptive and correlational analyses

In this sample, 25.70% (*n* = 220) participants reported that they had inflicted cyberbullying on other people in recent half year. The bivariate relationships among RFP, AFP, relatedness need satisfaction and cyberbullying perpetration were examined (see Table 1).

**Table 1:** Descriptive statistics.

Variables	1	2	3	4
1. Reciprocal filial piety	—			
2. Authoritarian filial piety	0.12^**^	—		
3. Relatedness need satisfaction	0.37^**^	−0.02	—	
4. Cyberbullying perpetration	−0.24^**^	0.05	−0.17^**^	—
*M*	4.38	2.40	5.54	0.33
*SD*	0.66	0.85	1.00	0.86
Skewness	−1.28	1.11	−0.83	3.61
Kurtosis	1.91	1.19	1.35	15.02

^**^p < 0.01.

### Testing for normal distribution

We tested whether data followed normal distribution. The skewness and kurtosis of RFP, AFP, and relatedness need satisfaction (see Table 1) fell within the acceptable range (i.e., skewness <|3|and kurtosis <|10|; Kline, 2016). However, the distributions of cyberbullying perpetration (skewness = 3.61, kurtosis = 15.02) were somewhat skewed. Thus, we used a square root transformation on the overall mean scores of cyberbullying perpetration to approximate the normal distributions. The transformed cyberbullying perpetration (skewness = 2.06, kurtosis = 3.52) was used for the following analyses.

### Linear regressions

Linear Regressions were adopted to examine how RFP and AFP uniquely predicted cyberbullying perpetration, with gender and age controlled. These multiple regression models and their main findings were summarized in Table 2. As shown in Table 2, RFP was negatively associated with cyberbullying perpetration (*β* = −0.19, *p* < 0.01), and AFP was positively associated with cyberbullying perpetration (*β* = 0.05, *p* < 0.05). These results suggested that RFP had a negative effect on cyberbullying perpetration and AFP had a positive effect on cyberbullying perpetration.

**Table 2:** Linear regression results.

	*β*	*t*
Gender	−0.20	−5.56^**^
Age	0.01	0.56
Reciprocal filial piety	−0.19	−7.30^**^
Authoritarian filial piety	0.05	2.43^*^
*R*^2^	0.11	

^*^p < 0.05, ^**^p < 0.01.

### Mediation analyses

**Figure 1:**
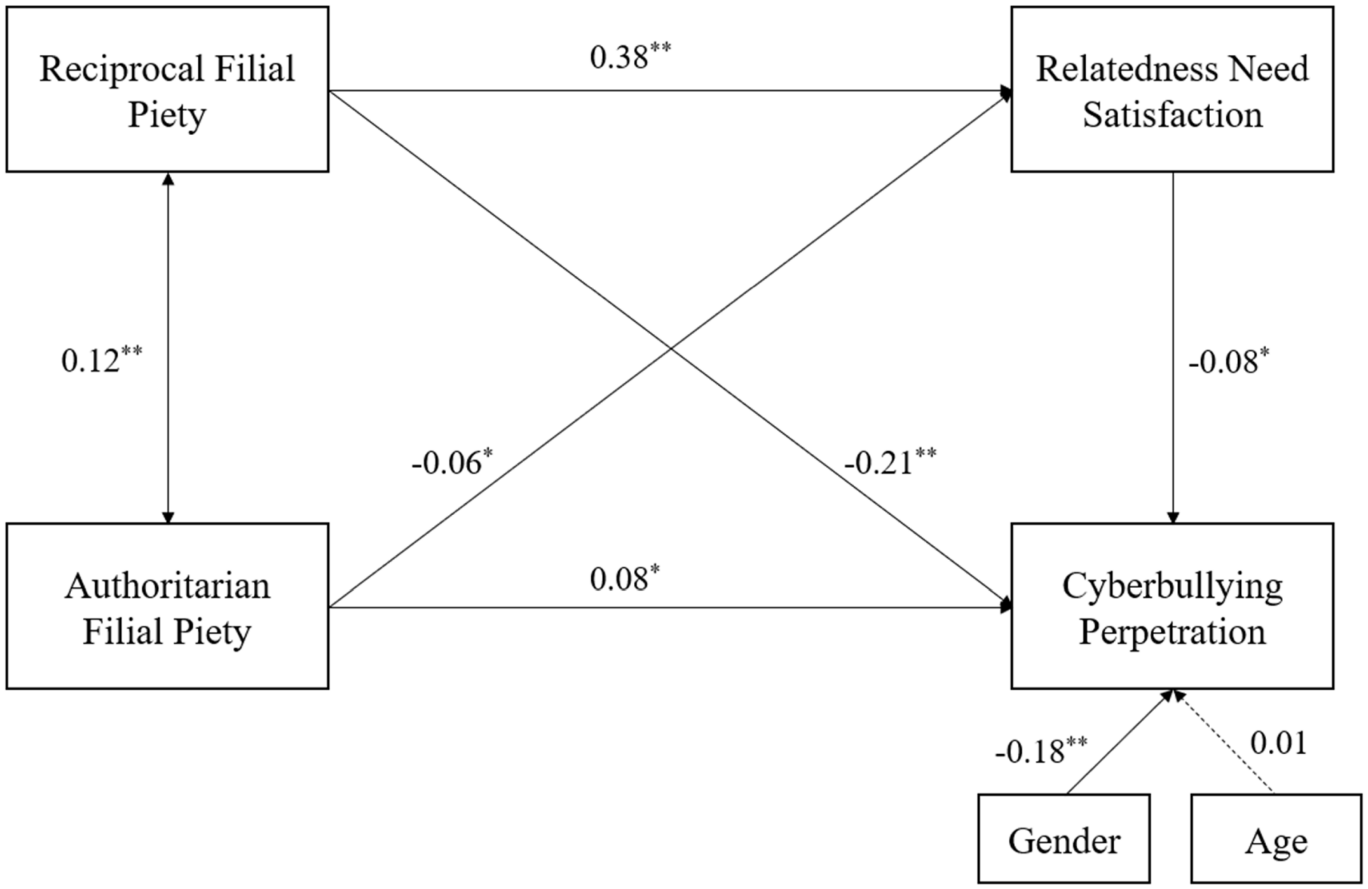
Structural model. *p < 0.05, **p < 0.01.

We tested our theoretical model using structural equation modelling (Figure 1). The results were as follows. RFP directly negatively predicted cyberbullying perpetration and AFP directly positively predicted cyberbullying perpetration. These results are consistent with H1. RFP and AFP impacted cyberbullying perpetration through the mediating effect of relatedness need satisfaction. RFP is positively correlated with relatedness need satisfaction, whereas AFP is negatively correlated with relatedness need satisfaction, and relatedness need satisfaction is negatively correlated with cyberbullying perpetration, which is consistent with H2. The fit indices of the mediational model were good, as follows: *χ^2^/df* = 4.28, CFI = 0.91, GFI = 0.99, IFI = 0.91, and RMSEA = 0.06.

## Discussion

### Filial piety belief and cyberbullying perpetration

The results provide a new position to understand the effect of family factors on cyberbullying perpetration by placing the topic within traditional Chinese family value. Although family influence on adolescents is a universal focus for global researchers, western psychologists have different emphasis from Chinese indigenous psychologists regarding the agency each member performs in a family. Western psychological theories constantly accentuate the importance of parents’ agency. For example, the attachment theory and the parental acceptable-rejection theory both encourage a healthy parenting style, or how parents behave influences children’s development and family environment (Bowlby, 1969; Rohner, 2004). Differently, indigenous psychology considers children’s agency to be more important. For instance, the dual filial piety model introduces a family structure where children’s action sets the keynote of family interaction and even influences their own psychological and behavioral adaption during their growth (Yeh and Bedford, 2003).

Specifically, previous studies examining the influence of family factors on cyberbullying perpetration mostly focus on parents’ agency, an established perspective adopted by western psychologists. For example, previous studies have found that more parents’ monitor (Ybarra and Mitchell, 2004), stronger parental support (Wang et al., 2009) and more negative attitudes parents have towards cyberbullying perpetration (Hinduja and Patchin, 2013) predict less children’s cyberbullying perpetration. However, the present study revealed that how children perceived their role in the family affected the odds of children perpetrating cyberbullying. The findings also revealed that different role perception of children in a family would have different influence on adolescents behavioral adaption. Children who believe that they are liable to take care of and love parents, holding RFP, will have less likelihood of cyberbullying perpetration. In contrast, children who are convinced by the social norm advocating parents’ absolute authority, holding AFP, will be more likely to perpetrate cyberbullying. These results suggest that both parents’ and children’s agency be considered in the future cyberbullying studies.

In addition, the results provide a possible interpretation for previous findings regarding the functioning mechanism between parenting and cyberbullying perpetration. We consider that parenting style may influence individual filial piety belief, given that individual endorsement of filial piety belief is the result of socialization, or that children are usually taught to accept this belief (Yeh and Bedford, 2003). Therefore, future research can explore whether parenting style impact on cyberbullying perpetration through children’s filial piety belief. Furthermore, past research has indicated that filial piety belief may moderate the effect of parenting on maternal control, perceived maternal support and mother–child conflicts (Wong et al., 2010). On that account, whether filial piety belief moderates the relationship between parenting and cyberbullying perpetration deserves further exploration.

Although this study sampled Chinese population, the results can inform cyberbullying studies in other eastern Asian and south-eastern countries such as Japan, Korea and Malaysia where cyberbullying has long been a critical social issue (Kim et al., 2019; Cho and Rustu, 2020; Rahman et al., 2020; Park et al., 2021). These areas are also deeply influenced by Confucius culture. Empirical studies have demonstrated that filial piety belief has a significant impact on young people from East Asia (Ismail et al., 2009; Nainee et al., 2022), suggesting that the present conclusion may be found in these countries.

Moreover, this study may provide a better understanding of cyberbullying in western society. Although filial piety as a concept does not exist in western, the legitimacy of parental authority and children’s attitude towards that share many similarities with filial piety regarding theoretical overtones and functioning mechanism. For example, both concepts designate role obligations to children in the family hierarchy. Children’s perceived parental authority can reduce parent–child conflicts, thus decreasing delinquency; parental legitimacy mediates the effect of parenting style on changes in delinquency (Zhang et al., 2006; Trinkner et al., 2012). Similarly, AFP significantly impacts on parent–child conflicts and children’s externalizing behaviors; AFP can act as a mediator on the association between parenting style and children’s life satisfaction (Li et al., 2014; Nainee et al., 2022). Given these similarities, we speculate that children’s perceived legitimacy of parental authority may influence cyberbullying perpetration. Recently, western researchers have revealed that the dual filial piety measure adapts well on a Polish sample (Różycka-Tran et al., 2021), communicating a future direction for researching cyberbullying perpetration in western cultural background.

### Mediation of relatedness need satisfaction

This mediation results contribute to the domain by adopting the self determination theory to illustrate how the two dimensions of filial piety belief impact differently on cyberbullying perpetration. The self determination theory argues that individual unsatisfied need is conducive to their aggressive behavior (Hein et al., 2015; Fousiani et al., 2016; Ryan and Deci, 2017), providing us a way of understanding the functioning mechanism of the dual filial piety model, which has barely been investigated in previous research. Our findings show that individuals who score high on RFP are more satisfied with their relatedness need than those who score high on AFP. Subsequently, according to the self determination theory, RFP holders may be less likely to perpetrate cyberbullying than AFP holders. The results empirically supported the self determination theory as well as bringing forth further application of this theory in relation to filial piety. Future researchers can study whether relatedness need satisfaction mediates the association between filial piety belief and other types of aggression, for instance, school bullying.

In consistent with the current findings, previous findings show that need satisfaction can be a mediator on the relationship between family factors and aggressive behavior. For example, Fousiani et al. (2016) found that need satisfaction mediates the relationship between parenting style, including psychological control and autonomy support, and cyberbullying perpetration. Choe and Read (2019) also found the mediating role of need satisfaction on the relationship between parenting and aggression. Although these results are congruent with our findings in the theoretical sense, previous studies focus on parents’ agency while we on children’s agency.

### Limitation

The current study has three methodological limitations. First, the data collection relied on self-reported method and the self-response bias tendencies exist. Nevertheless, we adopted several measures to deal with the problem, such as asking the participants to complete the questionnaire independently according to their own conditions, highlighting that their answers’ confidentiality, emphasizing that there was no wrong or right answer to the questions, using different format of questionnaires, and ensuring answers’ anonymity. Second, the current research completely relied on a sample of students from China mainland, so generalization of the findings might be limited. Finally, this study employs a cross-sectional design, and thus the results may be unreliable across time. Therefore, the effects of filial piety belief on cyberbullying perpetration should be examined further by using longitudinal or experimental design.

## Data availability statement

The raw data supporting the conclusions of this article will be made available by the authors, without undue reservation.

## Ethics statement

The studies involving human participants were reviewed and approved by the Research Ethics Committee of School of Educational Science, Xinyang Normal University. Written informed consent to participate in this study was provided by the participants.

## Author contributions

HW and ML designed the work. HW, LL, and ML collected the data, analyzed the data results, drafted the manuscript, and revised the manuscript. All authors contributed to the article and approved the submitted version.

## Conflict of interest

The authors declare that the research was conducted in the absence of any commercial or financial relationships that could be construed as a potential conflict of interest.

## Publisher’s note

All claims expressed in this article are solely those of the authors and do not necessarily represent those of their affiliated organizations, or those of the publisher, the editors and the reviewers. Any product that may be evaluated in this article, or claim that may be made by its manufacturer, is not guaranteed or endorsed by the publisher.
